# Experimental Music Workshop Facilitating Participant Transformation: Characteristics and Significance of Imus Music

**DOI:** 10.7759/cureus.107565

**Published:** 2026-04-23

**Authors:** Akiko Tajima, Junko Tanaka, You Wakao

**Affiliations:** 1 Occupational Therapy, Iro Sosei University, Iwaki, JPN; 2 Occupationl Therapy, Imus Music, Kurashiki, JPN; 3 Integrative/Complementary Medicine, Kobe University, Kobe, JPN

**Keywords:** experimental music, imus music, music therapy, personal transformation, workshop

## Abstract

This study aimed to explore the characteristics and potential significance of Imus Music, an open workshop in which everyone in experimental music can participate. Accordingly, qualitative and inductive analysis was conducted based on individual interviews with two participants and focus group interviews with two organizing musicians and an occupational therapist. Initially, the participants expressed self-denial and uncertainty about self-expression. However, their self-esteem improved with the progression of their involvement in the workshop. The musicians prepared meticulously to provide an enjoyable and heterogeneous experience. An occupational therapist implemented strategies to help the participants engage positively with the musicians’ objectives. Overall, the findings suggest that the significance of Imus Music lies in the distinctive features of its workshop environment and music, which facilitate participants’ engagement with experimental elements such as improvisation and contingency and enable them to experience a previously unfamiliar sense of freedom.

## Introduction

When examining the impact of music on humans, the historical development of music therapy and musical activities has focused on human wellbeing [1]. However, in recent years, the scope of such activities has expanded to the community, prompting a review of the relationship between therapists and recipients confined within therapy rooms. The forms of music used in this context emphasize reciprocity and intimacy, with a focus on the music's experimental elements [1]. This study examines the new musical activity known as Imus Music within the context of introducing experimental music to the community. The novelty of this study in its focus on the heterogeneity, contingency, and instability inherent in experimental music, developing individuals’ musical activities while also closely investigating its positive impact on them.

The experimental music used in community music activities is often nontechnical in nature and does not adhere to conventional musical techniques. As suggested by its name, experimental music is “experimental” in nature; that is, no effort is made to make it perfect [2,3]. According to one of the present article's co-authors and occupational therapist, Junko Tanaka*, *experiencing such a high degree of freedom can enhance the self-esteem of individuals with mental or developmental disabilities [4]. For the past 7 years, Tanaka has worked with two musicians having a background in experimental music to conduct experimental music workshops, “Imus Music,” the name having originated from a workshop that was conducted in a room next to a café called Imus Café.

Imus Music does not follow any particular model aimed at using music in treatments or therapeutic settings to promote personal transformation. Instead, it emphasizes the individual and affirms their existence by creating an experimental musical space that promotes free thinking, relinquishes preconceived notions of music, and guarantees free musical forms. In this manner, all participants can access and enjoy music and find meaning in it. Further, care is taken to ensure that organizers and participants remain on an equal footing and do not engage in curative or therapeutic relationships [4]. In other words, Imus Music explores the intersection between participation ethics and experimental music, enabling participants to gain meaning from musical activities [5]. From the perspective of musical activities, Imus Music intentionally includes elements that may surprise or occasionally cause discomfort to the participants, rather than emphasizing empathy and reassurance, which is the main focus of present-day music therapy [6].

However, initially, activities and policies were not decided in this manner. The process began with songwriting workshops, wherein participants wrote lyrics and musicians improvised melodies. Over time, experimental music was gradually incorporated into the process, finally resulting in the current format. Moreover, the workshop started including spontaneous activities catering to participants’ reactions, rather than being limited to music alone. Wakao [2] highlights the importance of including such experimental music and aleatoric activities, and Moorehouse [7] recommends the proactive use of experimental music in the workshop.

Although research indicates that Imus Music enhances participants’ self-esteem, motivation, and attachment to work [4], it does not clarify the following: (a) creation and development of the experimental music workshop by musicians, (b) sense of meaning and personal transformation achieved by participants through Imus Music, and (c) role of the organizing occupational therapist. To clarify these issues, we conducted two studies. The first study involved individual interviews with two participants, and the second study comprised focus group interviews with musicians and an occupational therapist. Our findings reveal the relationship between the objectives of this unique initiative and participants’ experiences and clarify the significance of and challenges posed by experimental music workshops.

In this study, “freedom” refers to a participatory condition in which participants can choose whether and how to express themselves without being constrained by predefined musical rules, external evaluation, or fear of failure. Similarly, “heterogeneity” refers to the workshop’s deliberate inclusion of unfamiliar, diverse, and non-standard musical and interpersonal elements, including improvisation, contingency, and multiple forms of participation.

## Materials and methods

Overview of Imus Music

This section provides an overview of Imus Music, with reference to Tanaka [4,8]. Imus Music began in 2017, with sessions being held once a month in a room of a church in Kurashiki, Japan. Two musicians with extensive experimental music expertise alternated as facilitators for participants; further, an organizing occupational therapist served as a sub-facilitator, creating an environment ensuring everyone’s free participation.

As of March 2024, 76 sessions had been held with 35 participants. The average number of participants in the most recent period (12 sessions from April 2023 to March 2024) was 12 to 13. Eight continuing participants participated five or more times a year, and the continuous participation rate was 65%.

The two musicians alternated in leading the sessions, and there was no predetermined format for the activities of each session. Facilitated by the leading musicians, these experimental music workshops were open to all participants and consistently provided experiences that challenged the preconceptions and traditional notions of music. For example, in one session, participants ventured into the streets to collect intriguing sound sources and, subsequently, examined them with the other participants. Further, lectures were provided to address participants’ self-doubt and foster a positive perspective in them. Table 1 depicts a segment of the program description.

**Table 1:** Example of an Imus Music program description.

Program	Description
Installation	Light, shadow, sound, paper, performance. With the lights being turned off and several papers hanging from the ceiling, the performance involved shadow puppets, free improvisation, and creative dancing using cellophane, plastic bottles, musical instruments, voice, and physical expression.
Songwriting	The theme was for G7 to coincide with the G7 summit. Participants came up with seven words beginning with the letter G, and everyone worked together to create lyrics based on those words. Finally, the musicians composed a melody, and everyone sang along.
Imus Song	Using only a piano’s black keys, participants chose one note at a time to create a basic musical pattern. The musicians composed songs based on this pattern, and participants choreographed dances in accordance with the music; in the end, everyone sang and danced together.
Soothing music	The musicians requested the lights to be turned off and instructed everyone to lie down on the floor or lean against a wall in a relaxed position and close their eyes. Participants improvised calm sounds using their favorite instruments or voices, and then listened to these sounds.
Mini lecture	Musician Mr. D gave a lecture on “anti-natalism.” The lecture was given after one participant said, “I wish I had never been born.” The meaning, types, and differences in the interpretation of anti-natalism were discussed from the perspectives of philosophy, religion, and literature. The lecture ended with Mr. D’s own denial of anti-natalism, to which the participants reacted with relief.

Study design

This study employed a two-stage qualitative design to clarify the characteristics and significance of Imus Music. Study 1 focused on how continuing participants described their experiences of participation and perceived personal transformation. Study 2 then examined the perspectives of the two facilitating musicians and the organizing occupational therapist, with particular attention to workshop preparation, facilitation, and participation support. The second study was intended to extend and contextualize the findings of Study 1 by exploring how the workshop environment and support practices may have contributed to the changes described by participants.

Study setting

Imus Music was held in a room in a church in Kurashiki, where interviews for both Study 1 and Study 2 were also conducted in a private room to ensure participants’ privacy.

Sampling and eligibility

Study 1 used convenience sampling among continuing Imus Music attendees. Individuals were eligible if they had participated continuously in Imus Music, were able to reflect on their experiences, and agreed to be interviewed. Individuals who did not provide consent or who were unable to participate in an individual interview were excluded.

Study 2 used purposive key-informant sampling. The occupational therapist organizer and two facilitating musicians were recruited because they were directly involved in planning, organizing, and conducting the workshop. Because this was an exploratory qualitative study, no formal sample size estimation was performed.

Participants

The aim of Study 1 was explained to the two participants who agreed to continuously participate in the study. Ms. A was in her 40s and had participated 10 times. Although she did not appear to have any disability, she considered herself a perfectionist. She needed a place to belong outside the workplace. Another participant, Ms. B, was in her 70s and had participated 37 times. She had no disabilities, and she participated following an introduction by Ms. C (an acquaintance). She had no specific needs at the start of her participation.

In Study 2, Ms. C, the occupational therapist who organizes Imus Music, and Messrs. D and E, the musicians who facilitate the event, participated. Ms. C specializes in music. Once she became too ill to play the piano, she learned improvisation under Mr. D and performed relevant research. Mr. D, who has experience and knowledge in improvisational music, told us retrospectively, “I have lived a life that rejects authority and people with authority.” Finally, Mr. E, who usually teaches gamelan (a traditional Indonesian ensemble that he studied at university), holds an “interest in sound and silence,” and conducts “workshops that tie into these themes,” participated in the study.

Study 1

Individual interview surveys were conducted in July 2023. The first author conducted the interviews and analyzed the interview data. The first author had no prior relationship with the interview participants. Being a qualitative researcher and instructor, the first author conducted the study as per the request of the second author, who was the organizer of Imus Music. The first author participated only once in Imus Music while conducting preliminary research for the interviews.

The individual, semi-structured interviews, which enabled us to focus on participants’ ongoing involvement and evolving self-perceptions, were conducted in a private room in the same church. The interview guide for Study 1 included questions about the circumstances surrounding participation and participants’ reasons for joining, the particularly memorable sessions and why, and perceived changes in their feelings before and after participation. Each interview lasted approximately 90 minutes.

**Figure 1:**
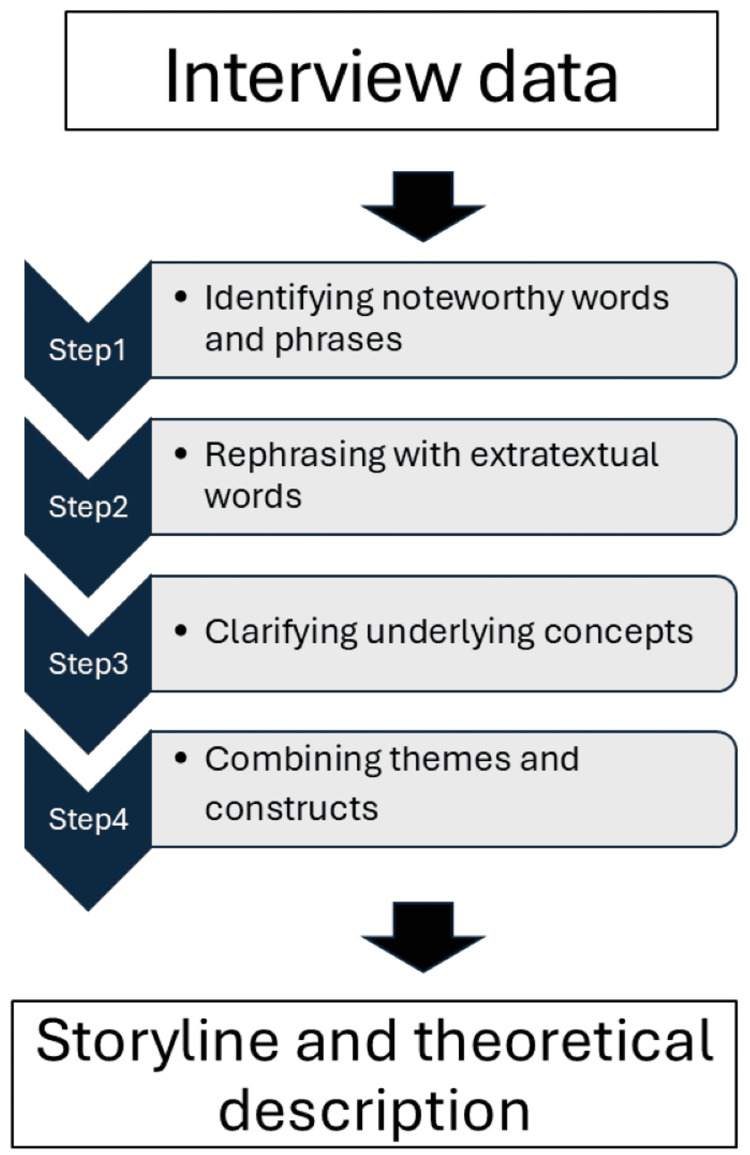
Flowchart of the SCAT analytical procedure. This figure illustrates the four-step SCAT process used in Study 1, from identifying noteworthy words and phrases to integrating themes and constructs, followed by storyline development and theoretical description. Figure created by the authors using PowerPoint (Microsoft Corporation, Redmond, USA) without generative AI. SCAT: Steps for Coding and Theorization

Before analyzing the collected data, all interview survey results were recorded, and the audio data transcribed verbatim. The resulting qualitative data were analyzed using the Steps for Coding and Theorization (SCAT) method. No statistical software or dedicated qualitative data analysis software was used in this study. After verbatim transcription, the interview data were analyzed manually by the first author using the SCAT. We used this method, popular across disciplines, including healthcare, human welfare, and education, because its analytical procedures are formal, explicit, and suitable for relatively small-scale data [9].The SCAT analysis procedure involved writing segmented data from verbatim transcripts into a dedicated format and, then, coding them in four stages: (1) identifying noteworthy words and phrases in the data, (2) indicating the extratextual words that rephrase the phrases, (3) clarifying the extratextual concepts that explain the words, and (4) combining the themes and constructs emerging from the previous stage. Subsequently, a storyline was written using all the themes and constructs obtained from the coding stages, and a theoretical description was created based on the storyline. Storylines are structures that recontextualize the meaning of the themes and constructs obtained from analysis results and, hence, are crucial in SCAT. Finally, theoretical descriptions were obtained by fragmenting the storyline. The SCAT analytical process used in this study is illustrated in Figure 1.

Study 2

Similar to Study 1, Study 2’s interviews and data analyses were conducted by the first author. Before the study, the first author had no prior acquaintance with Mr. E. In October 2023, focus group interviews were conducted with three participants. These interviews helped obtain qualitative information by applying group dynamics [10] and were considered an appropriate method for this study to clarify the similarities and differences among the three groups. The researchers clarified the purpose of the study to the participants before conducting the interviews and suggested appropriate topics pertaining to Study 1's results to encourage active discussion. The interviews lasted 90 minutes and were conducted in a private room in the same church.

Study 2 used a semi-structured focus group interview. The interview guide covered the preparation and objectives of the workshop, the characteristics of the sessions, interactions and relationships with participants, perceived participant changes, and the role of the occupational therapist within the workshop. Before analysis, the researchers recorded all interview survey results and transcribed the audio data verbatim to provide the second set of qualitative data.

Subsequently, Study 1's qualitative data were collected to identify participants’ changes before and after their participation in Imus Music and clarify the relevant session contents. Ms. C organized her own role, and Messrs. D and E organized their preparations, goals, and relationships with the participants. No statistical software or dedicated qualitative data analysis software was used in Study 2. After verbatim transcription, the focus group interview data were reviewed and organized descriptively by the first author in relation to the changes identified in Study 1.

Translation of interview data

All interviews in Studies 1 and 2 were conducted in Japanese. The interview data and selected quotations were professionally translated into English for manuscript preparation; however, back translation was not performed.

Rigor and trustworthiness

To enhance the rigor and trustworthiness of the study, the first author conducted all interviews and analyzed all qualitative data according to the procedures described above. In Study 1, data were analyzed inductively using SCAT, whereas in Study 2, the focus group data were reviewed and organized descriptively in relation to the findings of Study 1. Interview guides were used in both studies to support consistency in data collection. Preliminary interpretations were shared with participants in both studies for member checking, and no major discrepancies were identified. The reporting of this qualitative study was also refined with reference to the COREQ checklist to enhance transparency and completeness [11]. Because the first author conducted the interviews and analysis after limited prior exposure to the workshop, her positionality may have influenced data collection and interpretation; this possibility was taken into account during analysis.

Ethical considerations

In Studies 1 and 2, the second author explained the study’s aims to research participants and obtained their consent to participate before conducting research. Moreover, the studies were conducted after obtaining approval from the ethical review committee of the second author’s affiliated institution (Kawasaki University of Medical Welfare; approval number: 20-017).

## Results

Study 1 results

Results for Ms. A

The analysis yielded fifteen constructs and seven theoretical descriptions. Table 2 depicts a segment of the SCAT analysis for Ms. A.

**Table 2:** Segment of Ms. A’s steps for coding and theorization analysis.

Text	Notable words and phrases from the text	Paraphrasing words in the text	Extra-textual concepts that explain the text to the left	Theme and constructs
Interviewer 1: What originally motivated you to get involved? Speaker: I actually participated in the first Imus Music session, but at the time, it did not feel like a good fit. At the time, the slightly friendly atmosphere did not suit me. I felt it should have been a bit more edgy, more classroom-like, and I did not think it was a place where I could express myself. Interviewer 1: It seems like you came here to learn. Speaker: Yes, that’s right. I thought it was something like that and a little bit different. But then, during the COVID-19 crisis, I had no one to talk to, even at work. I live alone, so I would just go shopping and then come home. I even stopped keeping in contact with friends. Interviewer 1: So just work, and home, it’s as simple as that. Speaker: Yep, just back and forth between the two.	The friendly atmosphere did not suit me./I did not think it was a place where I could express myself./During the COVID-19 crisis, I did not have anyone to talk to.	I could not adapt to the atmosphere of being able to enjoyably connect with other participants./I did not think I would be asked to express myself./I did not have anyone to talk to during the COVID-19 crisis	Provides a friendly atmosphere for people who do not fit in./The surprise of being asked to suddenly express oneself./The unique loneliness of the COVID-19 crisis.	Participating in Imus Music allows for friendly connections./Feeling confused when asked to freely express oneself./Feeling truly lonely amid the COVID-19 crisis.
Interviewer 1: So, it ended up being like a round trip. Speaker: The days went on and on like that. It was only when I was all alone that I realized that I needed to connect with others. Up until then, I guess I thought that humans could survive on their own. But when the COVID-19 crisis occurred, and I was isolated from others, I was forced to really know what it means to be alone. I had been thinking about connecting with others in some way or another, and it was then that I discovered Imus Music, where I could go and enjoy a friendly atmosphere. At that moment, I really desired such a friendly atmosphere.	I needed to connect with other people./I really felt what it means to be alone./Imus Music is a possibility, and it has a friendly atmosphere./I wanted that friendly atmosphere.	Losing connections with people was hard./I felt true loneliness./By going to Imus Music, I could connect with other participants./I needed to experience a sense of fun and connection with others.	The pain of losing connections with people./Feelings of true loneliness./Imus Music is a place where you can make fun connections with people./A need for enjoyable connections with people.	Feeling truly lonely amid the COVID-19 crisis./Participating in Imus Music allows for friendly connections.
Interviewer 1: So, it ended up being like a round trip. Speaker: The days went on and on like that. It was only when I was all alone that I realized that I needed to connect with others. Up until then, I guess I thought that humans could survive on their own. But when the COVID-19 crisis occurred, and I was isolated from others, I was forced to really know what it means to be alone. I had been thinking about connecting with others in some way or another, and it was then that I discovered Imus Music, where I could go and enjoy a friendly atmosphere. At that moment, I really desired such a friendly atmosphere.	I needed to connect with other people./I really felt what it means to be alone./Imus Music is a possibility, and it has a friendly atmosphere./I wanted that friendly atmosphere.	Losing connections with people was hard./I felt true loneliness./By going to Imus Music, I could connect with other participants./I needed to experience a sense of fun and connection with others.	The pain of losing connections with people./Feelings of true loneliness./Imus Music is a place where you can make fun connections with people./A need for enjoyable connections with people.	Feeling truly lonely amid the COVID-19 crisis./Participating in Imus Music allows for friendly connections.

The storyline is as follows: Ms. A was proud that she had “overcome many hardships and become perfectly independent.” However, she felt “uneasy about sharing her thoughts with others.” She had a “preference for passive musical activities” and was invited to participate in Imus Music. She was under the misconception that, at Imus Music, activities were provided unilaterally; hence, she was confused by the workshop’s promotion of the freedom of self-expression and soon stopped participating. However, she became extremely lonely during the coronavirus disease 2019 (COVID-19) crisis and joined Imus Music to escape the loneliness. Imus Music is characterized by the improvisational nature of its programs and its promotion of free self-expression. Ms. A, a long-term participant, initially found Imus Music to be “a place where one can feel at home without being nervous even if it’s one’s first time” and “a place for fun interaction.” She told us that her participation in Imus Music enabled her to connect with friendly people. Because Imus encourages free self-expression, Ms. A was initially confused by the expectations surrounding free self-expression. However, she gradually realized her internal diversity and started recognizing changes within herself. Now, “what she really wants is true freedom without any restrictions.”

The storyline-based theoretical description is as follows:

A-1: Although Ms. A was proud that she had “overcome many hardships and become perfectly independent,” she was “uneasy about sharing her thoughts with others.” 

A-2: She had a “preference for passive musical activities,” and she was invited to participate in Imus Music. However, she was under the misconception that, at Imus Music, activities would be provided unilaterally; hence, she was confused by the workshop’s promotion of free self-expression and soon stopped participating. 

A-3: Ms. A became extremely lonely during the COVID-19 crisis and decided to join Imus Music to escape the loneliness. 

A-4: Imus Music is characterized by the improvisational nature of its programs and its promotion of free self-expression. 

A-5: Ms. A found Imus Music to be “a place where one can feel at home without feeling nervous even if it's one’s first time” and “a place for fun interaction.” Participating in Imus Music enabled Ms. A to connect with friendly people. 

A-6: Because Imus promotes free self-expression, she was initially confused by the expectation of free self-expression; however, she gradually became aware of her internal diversity and began recognizing changes within herself. 

A-7: Ms. A finally realized that she really wants true freedom without any restrictions. 

Results for Ms. B

The analysis yielded 10 constructs and six theoretical descriptions. The storyline is as follows:

Ms. B felt a deep-rooted sense of self-denial because she resented her parents for being selfish. However, she considered her life to be “blessed with good luck,” and was grateful to have built a good relationship with her daughter. The invitation to participate in Imus Music was an event in “a life blessed with good luck.” At Imus, she met a trustworthy teacher whose perspective on her existence and her music was positive. Moreover, she felt that “each participant’s musical expression is respected, which makes Imus Music pure fun.” She reported being filled with a sense of self-esteem and having built cordial relationships with the other Imus Music participants.

The storyline-based theoretical description was as follows:

B-1: Ms. B has a deep-rooted sense of self-denial because she resents her parents for being selfish. 

B-2: Reflecting on her life, Ms. B noted that she was “blessed with good luck” and felt grateful for her “good relationship with her daughter.” 

B-3: For Ms. B, the invitation to participate in Imus Music was an event in “a life blessed with good luck.” 

B-4: At Imus Music, she met a trustworthy teacher whose perspective of her and her music was positive. 

B-5: She clarified that “each participant’s musical expression is respected, which makes Imus Music pure fun.” 

B-6: Now, Ms. B is filled with a sense of self-esteem and has built cordial relationships with other participants. 

Study 2 results

The details of each individual’s comments on each topic are discussed in the following section.

Preparation

Both Messrs. D and E prepared for each session. Mr. D thought extensively about how to provide participants with a different musical experience each time by including activities that he personally enjoyed and felt others would enjoy as well. Mr. E thought about a fundamental program while considering his current interests, what he had enjoyed doing elsewhere, and the season. However, both Messrs. D and E said that they might make some changes depending on the situation.

The following exchange is excerpted from the focus group interview and illustrates how Mr. D and Mr. E understood the preparation required for each session. Q refers to the interviewer.

Mr. D: Mr. E said that he made several efforts in preparations, and I’m the same. Allowing people to encounter different things requires an incredible amount of preparation and delicacy. Allowing participants to experience different music each time requires a thoughtful approach. This can be very trying.

Q: I see. You even seem to be out of sorts today. 

Mr. D: Yes, I’m trying really hard to think of something participants wouldn’t have done before and that I really enjoy and that they’d enjoy, as well.

Mr. E: I’ve been getting ready for quite some time now. I’m thinking about what to do. Today, Mr. D is in charge. I’m trying to think of something to do once he’s done and it’s my turn. I usually plan my performance in advance, thinking about what is currently popular, things that I have enjoyed doing elsewhere, or the season, but sometimes, when I actually present it in front of everyone, it just doesn’t feel right; so, I might tweak the program a bit.

Q: Right on the spot?

Mr. E: Yep, I make some changes right on the spot. I try to put a lot of thought into the theme. What it should consist of, and so on. As the day approaches, I get a little nervous, wondering if everything will be okay.

Intention

Regarding his intentions, Mr. D hoped that participants would be visibly cheered and not become dull on receiving an opportunity to “encounter something completely different.” In response to Mr. D’s comment, Mr. E stated that he had no specific expectations, saying, “I haven’t thought about it,” and that he wanted participants to “have fun and do something thrilling.” He found it amazing how the participants, who supposedly had no experience performing on stage, gradually “created space” and “performed brilliantly.” Accordingly, we found no differences between the two musicians in the strength of their intentions and their perceptions of the participants’ positive changes.

Mr. D: Because it’s all about empathy, everyone just moans, and that’s the end of it. All therapy is like that; so, it’s pointless. It’s much more meaningful to be confronted by something new and be forced to think about it. I wasn’t originally thinking about doing something like this, but I wanted to do something different, by using empathetic songs and getting everyone to show their emotions, and Mr. E is really good at creating unique presentations. Just today, I was wondering why everyone looked so bright and cheerful, and then I realized it was because they were doing something they had never done before. This feeling has grown stronger and stronger as I have continued doing it, but I don’t know if Mr. E feels the same way. 

Mr. E: I really don’t think much about it. I don’t really think about what will happen to everyone if I do something. I don’t really think about whether it will give them some sense of freedom or anything. I try to just have fun with everyone there and provide them with a little excitement. I don’t really get into the details of what the end result will be by saying stuff like, “If you do X, Y will happen.” I don’t think most participants normally stand on stage and give presentations. We create a space where such people can make sounds, sing, or even dance on their own. Everyone can do this brilliantly. Everyone is doing something amazing.

Participant relationships

In Study 1, Mr. D spoke about the changes in Mses. A and B. In response to the normative thinking (B-1) that is the source of Ms. B’s suffering, she is told that “many people think the same way, and that’s not bad” and that she should continue to “let it loose.” According to Mr. D, Ms. A’s confusion on being asked to express herself (A-2) was caused by the norms that restricted her self-expression, and thinking proactively about freeing oneself from this confusion is a practice for “removing constraints.” Mr. E wanted to create a system that enables participants to act “spontaneously” and create a “space where people can concentrate and participate”; he says, “It doesn’t feel right when people are left out.” Accordingly, he advises participants to “just give it a try” and be happy if all goes well.

Mr. D: Suddenly, and quite unexpectedly, someone blurted out for the first time that it would have been better if they had never been born, and they said it so confidently that it shocked me, and I wasn’t even really sure what they had said. One has to really think about how to best respond to such a person. That’s why I want to continue to identify the things that cause such people to stumble and help them avoid this kind of misery.

Mr. D: I lectured everyone about how anti-natalism [the notion that childbirth is unethical] is a stupid idea and why we should abandon it. However, to be able to do this, I had to read about 10 books and do some research.

Mr. D: They say that they are expected to express themselves, but we certainly don’t hold ourselves to that standard. The reason such expressions are used is that they are based on an input of how something should be done and, so, simply express being forced to do something that doesn’t meet those standards. So, I think that just going home and thinking about how to think and what to do in this situation is extremely good practice.

Mr. E: This person said this before; I wonder about now? Maybe now, they would like to give it a try? If I speak up, and it goes over well, it makes me happy, and I think, “We did it!” So, I guess I’m interested in seeing the before and after in the person.

Mr. E: What I particularly value is voluntary participation. I want to create a system where participants can discover things on their own, set things up with their own two hands, and participate in the process.

Q: Is there any particular reason for that?

Mr. E: It’s partially because it’s easier for me to do it that way. I don’t like it when someone is left behind. It’s really difficult. I like it when everyone is really into it and participating to their fullest capacity; then, I can just kind of lean back and take it easy. In the end, I’ve found that it is better to work together than to try to handle it all on my own.

Role of the occupational therapist

In the workshop, the role of the occupational therapist, Ms. C, who served as the organizer, was to “not get involved in therapeutic matters” and “leave facilitation to the musicians.” Simultaneously, Ms. C undertook a subleader’s role, speaking to those who seemed out of place and actively involving herself in collaborative activities. In other words, Ms. C ensured that participants could share the musicians’ goals positively. In addition, she played the role of management in all aspects.

Ms. C: The division of roles is fairly clear, and I do not get involved in therapeutic matters. I leave it up to the musicians to facilitate this event, and although I do provide support, I basically act as a caretaker.

Q: And what does a caretaker do?

Ms. C: Sets up time for activities, makes reservations, keeps in contact with everyone, keeps a blog, and so on. The caretaker acts as a subleader. Basically, I leave it to the leader to proceed; but, for example, I’ll check to see if someone is interested in participating, or if there’s someone I’m interested in, I’ll talk to them, or if we decide to all sing together, I’ll take the initiative and sing along.

## Discussion

Imus Music space’s characteristics as seen through changes in participants

The Imus Music environment’s characteristics were examined based on the dialogue between participants A and B on their changes after participating in Imus Music in Study 1 and the preparation, aims, and participant relationships of facilitating musicians D and E in Study 2. First, Ms. A felt “uneasy about sharing her thoughts with others” (A-1) and had a “preference for passive musical activities” (A-2). While participating in Imus Music, she was confused by the promotion of freedom of self-expression and soon stopped participating (A-2). However, she became lonely during the COVID-19 crisis and decided to rejoin Imus Music to escape the loneliness (A-3). Then, Ms. A felt right at home at Imus Music and found it a place where everyone got along (A-5). Through repeated participation and self-expression, she gradually became aware of the diversity within herself and started recognizing changes within herself (A-6).

Despite “continuing to resent her parents for being selfish” and “having a deep-rooted sense of self-denial” (B-1), Ms. B started participating in Imus Music after a chance encounter (B-3). Her participation enabled Ms. B to meet trustworthy teachers who positively accepted her existence (B-4). Further, she found that “each participant’s musical expression being respected makes Imus Music thoroughly enjoyable” (B-5). Now, Ms. B is filled with feelings of self-esteem (B-6) and, in hindsight, considers her life to have been blessed with “good luck,” as exemplified by her participation in Imus Music.

Regarding the “preparation” involved in Study 2, musicians Messrs. D and E, the workshop’s facilitators, carefully decided on and prepared activities so that participants could have fun and new experiences in each session. Mr. D hoped that participants would feel awake and refreshed on encountering something different from the everyday. To improve his “relationships with participants,” Mr. D aimed to “remove the constraints” that made the participants feel rejected or constrained. Contrastingly, Mr. E expected the participants to enjoy themselves more spontaneously. Therefore, by preparing and implementing mechanisms for spontaneous movement in experimental music, the musicians enabled the participants to discover their self-expression and self-understanding and freed them from the restrictions placed by social values ​​and norms.

Both Mses. A and B went from having a “deep-rooted sense of self-denial” to feeling “fulfilled with self-approval” and from feeling “confused by the expectation of free self-expression” to becoming “aware of the diversity within themselves.” Accordingly, it seems that the musicians’ preparation succeeded and they realized their aims and, as intended by occupational therapist C, the Imus Music environment enabled participants to free themselves from self-deprecating social values, norms, and restrictive self-perceptions; affirm their existence; and realize their freedom of self-expression.

However, Ms. A initially felt confused by the request to express herself freely and withdrew from the group (A-2). Similarly, several other participants may initially feel confused when asked to freely express themselves musically. This may be the reason why some participants dropped out of the workshop. According to Mr. E, “It doesn’t sit well with me when someone has been left out.” Hence, he devised a way to remove the barriers to self-expression by establishing a rule that only the black keys be played (see Imus Song in Table 1). Meanwhile, Mr. D’s compositions included familiar melodies (see Songwriting in Table 1); accordingly, the songs were easy for everyone to sing. Moreover, occupational therapist C spoke to the people who seemed unfamiliar with this approach to ensure that the participants could share the musicians’ objectives positively. Such foresight was very effective and, although Ms. A stopped participating due to her feelings of confusion, she described the venue as “a place where one can feel at home without getting nervous even if it’s one’s first time” and “a place for fun interactions.” Currently, the high rate of continued participation suggests that musicians’ consideration helps participants to overcome any confusion to enjoy themselves, since Imus Music is a place where interaction helps alleviate loneliness. Table 3 summarizes the initial states and post-participation changes observed in Ms. A and Ms. B through their participation in Imus Music.

**Table 3:** Illustrative summary of participants’ transformations through participation in imus music.

Participant	Domain	Before participation	After participation
Ms. A	Self-expression	Confused by expectation of free self-expression	Became aware of internal diversity
	Belonging	Needed a place to belong outside the workplace	Found Imus Music to be a welcoming place for interaction
Ms. B	Self-regard	Deep-rooted self-denial	Became filled with self-esteem
	Social meaning	Carried longstanding resentment and pain	Met trustworthy teachers and built cordial relationships

Although the occupational therapist did not assume a traditional therapeutic role, her involvement was important in supporting participation at both interpersonal and organizational levels. Interpersonally, she observed participants’ readiness, spoke to those who appeared uncertain, and facilitated entry into shared activities without imposing participation. Organizationally, she maintained the structure necessary for the workshop to continue, including time management, reservations, communication, and coordination. From an occupational therapy perspective, these strategies can be understood as environmental structuring and participation support rather than direct therapeutic intervention [12]. This indirect but intentional involvement may have helped participants engage safely and voluntarily in an unfamiliar and potentially unsettling musical space.

**Figure 2:**
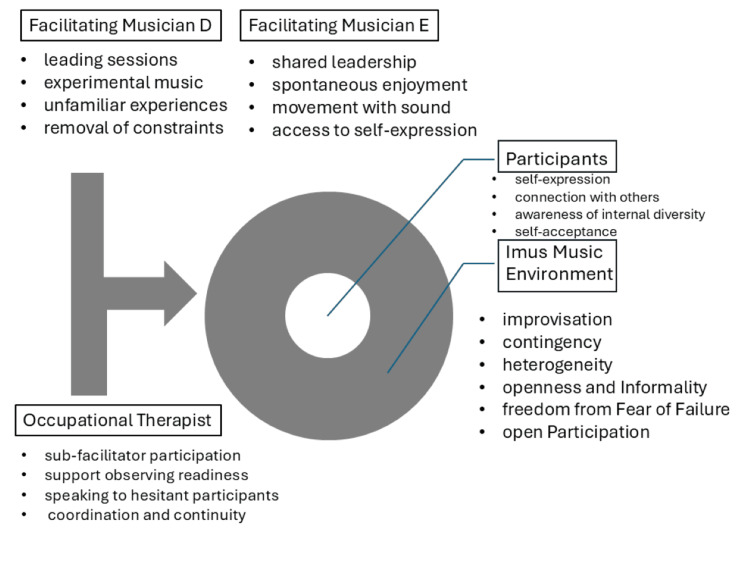
Schematic representation of the Imus Music framework. The figure illustrates the relationships among the alternating facilitating musicians, the occupational therapist as a sub-facilitator, the permissive workshop environment, and participants’ opportunities for self-expression and interaction. Created by the authors using Microsoft PowerPoint (Microsoft Corporation, Redmond, USA) without generative AI.

As interpreted from the findings, Figure 2 illustrates the overall framework of Imus Music, highlighting the relationships among the facilitating musicians, the occupational therapist, and the participants, as well as the conditions that supported participation and self-expression.

Imus Music features

The changes in Mses. A and B were due to the characteristics of the Imus Music venue and could be heavily influenced by the music played at the venue. Although the Imus Music program is developed based on music (Table 1), it can be distinguished from general musical activities by its attempts to enhance creative expression beyond the scope of music and focus on physical sensations. Despite being avant-garde, surreal, or punk sometimes, it is collectively called “experimental music.”

The most distinctive feature of Imus Music is its “different” quality compared to the “familiar music” emphasized by traditional musical activities. This is evidenced by Mr. D’s comment that he wants to provide an opportunity for participants to “encounter something completely different.” Music therapy emphasizes the “iso-principle” [6]; however, the musicians’ comments indicate that Imus Music deliberately avoids the emphasis on empathy and caring that characterizes homogeneous music and client-centered therapy [13]. People feel moved upon encountering something that they have never experienced in their daily lives. This can be unsettling but also exhilarating as a new experience.

In addition, Imus Music does not involve the discipline typical of Western music. The Imus framework highlights “loose,” “casual,” and “anything goes.” Accordingly, constraints are loosened before the program so that participants find it easy to remove them. Moreover, there are no mistakes in Imus Music. Mistakes are not consciously avoided; rather, the nature of the musical style indicates no possibility of the occurrence of mistakes. Ms. B’s “pure fun” and “full of self-affirmation” and Ms. A’s “awareness of one’s own diversity” are feelings arising from a warm and secure environment where the fear of failure is absent. The Imus Music approach does not clarify that there is a correct performance (or that all performances are correct); even if participants have no musical knowledge or skill, they can express themselves of their own accord or not at all without any fear of failure.

Significance of Imus Music

Imus Music ensures that participants experience an unprecedented sense of freedom by uniquely engaging in improvisational performances and experimental endeavors that emphasize chance. Unlike the familiar concept of singing, these activities involve the participants’ acquisition of freedom by coming into contact with unfamiliar and diverse concepts, which are probably linked to the liminality observed by cultural anthropologist Turner [14]. According to Turner [14], the concept of liminality refers to the boundary between consciousness and unconsciousness and between the everyday and extraordinary, indicating that a state of liminality can destroy customs and fixed lifestyles. Further, music therapist Ruud [15] uses this theory to discuss the significance of improvisation in music therapy. Moreover, co-author and occupational therapist Tanaka [16] emphasized the importance of liminal activities as follows: “Everyday entertainment can bring a certain sense of security and enjoyment to people’s hearts, but it does not provide the kind of deep-seated motivation that motivates people from the bottom of their hearts.” In other words, an encounter with the unknown facilitates the easy transcendence of consciousness and enables one to encounter the unknown self.

Simultaneously, liminality can cause such activities to force participants into an untenable position. Some people, such as Ms. A, gradually became accustomed to the program as they participated; however, some individuals might have discontinued the program after finding its inherent instability shocking. From a therapeutic perspective, such instability is undesirable and avoidable. However, according to the current study’s results, this instability was the factor that brought about the changes in Mses. A and B.

Thus, the findings of this study can be interpreted through Turner’s concept of liminality [14]. For example, according to the SCAT results for Ms. A, participation in Imus Music involved moving beyond familiar, passive forms of musical participation into an uncertain yet permissive space characterized by improvisation, contingency, and the absence of failure. Her initial confusion, followed by an emerging awareness of her own internal diversity, suggested a transitional process in which established assumptions about the self and about participation were temporarily destabilized. From this perspective, the significance of Imus Music may lie in its capacity to create a liminal space that enables new forms of self-understanding and connection with others. At the same time, it should also be noted that such instability may be challenging for some participants.

Imus Music embodies a novel approach that intentionally incorporates “encounters with heterogeneity,” a methodology that is outside the purview of existing music-based therapy programs and can provide participants with a sense of self-liberation and self-affirmation that is difficult to obtain using existing music therapies. When considered a community music activity, the program risks becoming a device for “culturally adapting” clients due to its assumption of community participation [1]. However, the undeniable novelty of Imus Music is that it is an initiative to gain freedom from a cultural perspective.

Limitations and future directions

This study has several limitations. First, the participant sample was very small, consisting of interviews with only two participants, which limits the broader transferability of the findings. Second, the study focused on a single community-based experimental music workshop, and the findings may therefore reflect the specific characteristics of Imus Music rather than those of experimental music workshops more generally. Third, the analysis was based on qualitative interview data from participants, musicians, and an occupational therapist, and the interpretation of change relied on retrospective accounts rather than direct measurement. In addition, the perspectives of individuals who may have discontinued participation or experienced the workshop negatively were not sufficiently represented. Future studies should include larger and more diverse samples, multiple sites, and, where appropriate, longitudinal or mixed-method approaches to further examine how and under what conditions experimental music may support self-expression, self-understanding, and community participation. Fourth, because Imus Music is designed as an open and flexible workshop format, exact replicability in the strict sense is inherently limited. Fifth, because this study involved a selective sample of continuing participants and was conducted in close proximity to the intervention context, there is a potential risk of interpretive overreach. The findings may primarily reflect positive experiences and perspectives, and the researcher’s positionality may have influenced data collection and interpretation. Therefore, the results should be interpreted as context-specific and exploratory.

This study highlights the characteristics and significance of Imus Music by examining the results of individual interviews with its participants, musicians, and occupational therapists. Future studies must further examine the positive impact of experimental music’s heterogeneity and provide a new approach for existing treatments and therapies.

## Conclusions

This qualitative study suggests that experimental music workshops such as Imus Music may provide a distinctive community-based space in which participants experience self-expression, self-acceptance, and connection with others. The findings indicate that the value of this approach may lie not only in the heterogeneity, improvisation, and contingency of the musical experience, but also in the creation of a permissive environment in which there is little fear of failure and participation is carefully supported by musicians and an occupational therapist. However, because such unfamiliar and unstable experiences may also be challenging for some individuals, further research with larger and more diverse samples is needed to clarify for whom and under what conditions this approach may be meaningful.
